# Tumour formation by single fibroblast growth factor receptor 3-positive rhabdomyosarcoma-initiating cells

**DOI:** 10.1038/sj.bjc.6605407

**Published:** 2009-11-03

**Authors:** M Hirotsu, T Setoguchi, Y Matsunoshita, H Sasaki, H Nagao, H Gao, K Sugimura, S Komiya

**Affiliations:** 1 Department of Orthopaedic Surgery, Graduate School of Medical and Dental Sciences, Kagoshima University, 8-35-1 Sakuragaoka, Kagoshima 890-8520, Japan; 2 Faculty of Engineering, Department of Bioengineering, Kagoshima University, 1-21-40 Korimoto, Kagoshima 890-0065, Japan

**Keywords:** rhabdomyosarcoma, sarcoma-initiating cells, cancer stem cell, fibroblast growth factor receptor 3 (FGFR3)

## Abstract

**Background:**

The hypothesis that malignant tumours are generated by rare populations of cancer stem cells that are more tumourigenic than other cancer cells has gained increasing credence. The objective of this study was to identify and characterise a subpopulation of human sarcoma-initiating cells.

**Methods:**

We examined established rhabdomyosarcoma cell lines by flow cytometry. Tumourigenesis was examined by xenograft models. Real-time PCR and immunohistochemistry were performed to examine the gene expression using cell lines and biopsy specimens.

**Results:**

Rhabdomyosarcoma cell lines included small populations of fibroblast growth factor receptor 3 (FGFR3)-positive cells. FGFR3-positive KYM-1 and RD cells were more strongly tumourigenic than FGFR3-negative cells. In addition, xenoengraftment of 33% of single FGFR3-positive KYM-1 cells yielded tumour formation. Stem cell properties of FGFR3-positive cells were further established by real-time PCR, which demonstrated upregulation of undifferentiated cell markers and downregulation of differentiation markers. We showed that in the absence of serum, addition of basic fibroblast growth factor maintained and enriched FGFR3-positive cells. On the other hand, ciliary neurotrophic factor reduced the proportion of FGFR3-positive cells. Real-time PCR and immunohistochemical examination revealed that embryonal rhabdomyosarcoma patient biopsy specimens were found to over-express FGFR3.

**Conclusions:**

Our findings suggest that rhabdomyosarcoma cell lines include a minor subpopulation of FGFR3-positive sarcoma-initiating cells, which can be maintained indefinitely in culture and which is crucial for their malignancy.

The hypothesis that malignant tumours are generated by rare populations of tumour-initiating cells (TICs), also called cancer stem cells, that are more tumourigenic than other cancer cells has gained increasing credence (Clarke and Fuller, 2006). TICs were initially identified in acute myeloid leukaemia (AML), and were found capable of inducing AML in immunodeficient mice (Lapidot *et al*, 1994; Bonnet and Dick, 1997). TICs have since been identified in numerous other tumours, including melanoma, lung, head, neck, pancreatic, prostate, colon, squamous cell cancers, and benign tumours (Collins *et al*, 2005; Fang *et al*, 2005; Kim *et al*, 2005; Dalerba *et al*, 2007; Prince *et al*, 2007; Loebinger *et al*, 2008; Xu *et al*, 2009). Although the AML TICs resemble and probably originate from the transformation of a stem cell, it is possible that other TICs originate from transformation of early or late progenitor cells. Thus, the definition of a TIC is not related to the cell of origin for a tumour but rather to its ability to self-renew, initiate cancer, and give rise to more differentiated cells that have lost the capacity for self-renewal and tumourigenic potential. The notion that cancer is driven by TICs has obvious therapeutic implications (Al-Hajj *et al*, 2004; Raguz and Yague, 2008). The efficacy of tumour response to systemic therapy has traditionally been assessed based on the bulk of tumour cells by monitoring of changes in tumour size (Therasse *et al*, 2000). However, if only a small fraction of TICs are capable of initiating cancer, then curative therapy should be designed to target these rare TICs rather than the bulk of nontumourigenic cells. Analysis of TICs might thus yield novel therapeutic targets.

In this study, we attempted to identify rhabdomyosarcoma-initiating cells (RICs) using cell surface markers. We examined many markers of undifferentiated cells. We found that human rhabdomyosarcoma cell lines include a small proportion of fibroblast growth factor receptor 3 (FGFR3)-positive cells. Single FGFR3-positive cells have the potential for tumour formation *in vivo*. In addition, tumours formed by FGFR3-positive cells could be used for serial propagation of tumours in animals. Moreover, basic fibroblast growth factor (bFGF) could maintain and enrich FGFR3-positive RICs in the KYM-1 cell line in the absence of serum. The easy method of preparation we describe will be useful for the development of anti-RICs target therapy.

## Materials and methods

### Cell culture

KYM-1 and RD rhabdomyosarcoma cell lines were purchased from Health Sciences Research Resources Bank (Tokyo, Japan). The A204 cell line was purchased from ATCC (Manassas, VA, USA). These cells were cultured in DMEM, supplemented with 10% FCS, 100 units per ml penicillin G, and 100 μg ml^−1^ streptomycin (Invitrogen, Carlsbad, CA, USA). In some experiments, KYM-1 cells were cultured in serum-free S-Clone (Sanko Junyaku, Japan) containing 10 ng ml^−1^ bFGF, 10 ng ml^−1^ epidermal growth factor (EGF), 2.5 ng ml^−1^ transforming growth factor beta (TGF-*β*), 10 ng ml^−1^ ciliary neurotrophic factor (CNTF), 10 ng ml^−1^ platelet-derived growth factor (PDGF)-AA, 10 ng ml^−1^ PDGF-BB, and 5 *μ*g ml^−1^ heparin. Normal human skeletal muscle cells (HSkMC) were purchased from TOYOBO (Osaka, Japan). HSkMC were cultured in skeletal muscle cell growth medium (Cell Applications Inc., San Diego, CA, USA) or DMEM, supplemented with 10% FCS, 100 units per ml penicillin G, and 100 *μ*g ml^−1^ streptomycin (Invitrogen). In all experiments, cells were maintained in 100 mm culture dishes (Nunc, New York, NY, USA) at 37°C in a humidified 5% CO_2_ per 95% air atmosphere.

### Flow cytometry analysis and sorting

Fluorescence-activated cell sorting analysis was performed using an Epics Altrta (Beckman Coulter, Fullerton, CA, USA). Cells were conjugated with anti-FGFR3 antibody (R&D, Minneapolis, MN, USA) for 30 min on ice. Cells were washed three times in PBS, re-suspended in the same buffer at 5 × 10^6^ per ml, and kept on ice until analysis. Live single cells (fixed FSC-A/FSC-W ratio; PI negative) were gated for analysis.

### Rhabdomyosarcoma patients' biopsy specimens

We obtained two biopsy specimens of human rhabdomyosarcoma from primary lesions. Pathological examination revealed that patient 1 had embryonal rhabdomyosarcoma and patient 2 had alveolar rhabdomyosarcoma. Biopsy was performed before chemotherapy or radiotherapy to make the diagnosis. Control muscle was obtained from surgery for scoliosis. The study protocol was approved by the institutional review board of the Kagoshima University. All patients and controls gave written informed consent.

### Real-time PCR

Each sample was run minimally at three concentrations in triplicate. All primer sets amplified 100–200 bp fragments. Primers were designed by Primer3. Total RNA was extracted using the miR-Vana RNA isolation system (Ambion, Austin, TX, USA) or TRIzol (Invitrogen). Reactions were run using SYBR Green (Bio-Rad, Hercules, CA USA) on a MiniOpticon machine (Bio-Rad). The comparative Ct (ΔΔCt) method was used to determine fold change in expression using *βII-macroglobulin*, *ACTB*, or *GAPDH*. The following primers were used: *CD34*: 5- CACCCTGTGTCTCAACATGG-3, 5-GGCTTCAAGGTTGTCTCTGG-3; *PAX3*: 5- GCCTGACGTGGAGAAGAAAA-3, 5-GCCTCCTCCTCTTCACCTTT-3; *PAX7*: 5-GAACCTGACCTCCCACTGAA-3, 5-CCTCTGTCAGCTTGGTCCTC-3; *MYF5*: 5-AATTTGGGGACGAGTTTGTG-3, 5- CATGGTGGTGGACTTCCTCT-3; *NANOG*: 5-AATACCTCAGCCTCCAGCAGATG-3, 5-TGCGTCACACCATTGCTATTCTTC-3; *OCT-4*: 5-GAGAACCGAGTGAGAGGCAACC-3, 5-CATAGTCGCTGCTTGATCGCTTG-3, *SOX2*: 5-AGAACCCCAAGATGCACAAC-3, 5-CGGGGCCGGTATTTATAATC-3; *MYH1*: 5-GCTCATCGAGAAGCCTATGG-3, 5-CAAAGAGAAGTGGGCCTCAG -3; *desmin*: 5-CATCGCGGCTAAGAACATTT-3, 5-GCCTCATCAGGGAATCGTTA-3; *myogenin*: 5-TGGGCGTGTAAGGTGTGTAA-3, 5-CGATGTACTGGATGGCACTG-3; *dystrophin*: 5-ACCACCTCTGACCCTACACG-3, 5-GCAATGTGTCCTCAGCAGAA-3; *β2-microglobulin*: 5-TCAATGTCGGATGGATGAAA-3, 5-GTGCTCGCGCTACTCTCTCT-3; *ACTB*: 5-AGAAAATCTGGCACCACACC-3, 5-AGAGGCGTACAGGGATAGCA-3; *GAPDH*: 5-GAAGGTGAAGGTCGGAGTC-3, 5-GAAGATGGTGATGGGATTTC-3.

### Immunohistochemical examination

We obtained two biopsy specimens of human rhabdomyosarcoma from primary lesions. Pathological examination revealed that patient 1 had embryonal rhabdomyosarcoma and patient 2 had alveolar rhabdomyosarcoma. Anti-FGFR3 (diluted 1 : 200, R&D) was used as a primary antibody. Rhodamine-conjugated donkey anti-rabbit IgG antibody (diluted 1 : 200; Chemicon, Billerica, MA, USA) was used as a secondary antibody. The cells were counterstained with Hoechst 33258 to identify nuclei. Immunohistochemistry with second antibody alone without primary antibody was performed as a control.

### Animal experiments

KSN/SLC nude mice were purchased from SLC. FGFR3-positive cells were collected by magnetic sorting by MACS according to the manufacturer's recommendations (Miltenyi Biotec, Gladbach, Germany). The following antibodies were used: PE-conjugated anti-FGFR3 antibody (R&D) and anti-PE Microbeads (Miltenyi Biotec). Cell inoculation was performed as reported earlier (Tanaka *et al*, 2009). Cells were mixed with a collagen gel, and were inoculated subcutaneously in 5-week-old nude mice. Grafts were excised and small portions of tumour (20 mg) were serially inoculated into other nude mice. In addition, graft was excised and trypsinised. Each number of cells was serially inoculated into other mice. Grafts were fixed with 10% buffered formaldehyde and stained with hematoxylin and eosin. All experimental procedures were performed in compliance with the guiding principles for the Care and Use of Animals described in the American Journal of Physiology and with the Guidelines established by the Institute of Laboratory Animal Sciences, Faculty of Medicine, Kagoshima University. All efforts were made to minimise animal suffering, to reduce the number of animals used, and to use possible alternatives to *in vivo* techniques.

## Results

### Rhabdomyosarcoma cell lines include a small portion of FGFR3-positive cells

**Figure 1:**
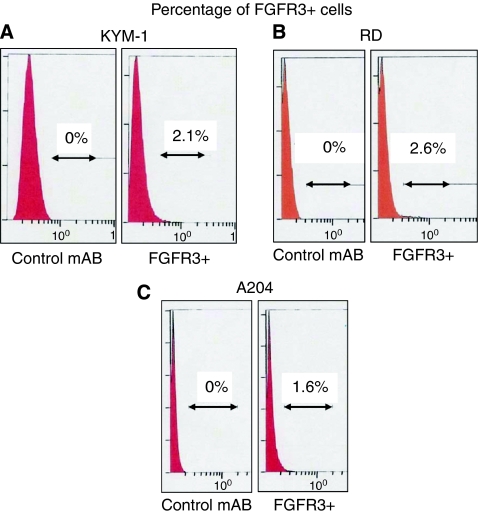
Rhabdomyosarcoma cell lines include a small portion of FGFR3-positive cells. Cells of human KYM-1 rhabdomyosarcoma (**A**), human RD rhabdomyosarcoma (**B**), and human A204 rhabdomyosarcoma (**C**) cell lines were labelled with anti-FGFR3 antibody and then analysed by flow cytometry. These three human rhabdomyosarcoma cell lines included small subpopulations of FGFR3-positive cells. These experiments were repeated at least three times with similar results.

To determine whether any of the established osteosarcoma and rhabdomyosarcoma cell lines included small portions of undifferentiated cell marker-positive cells, we performed flow cytometry. We examined many markers of undifferentiated cell, such as side population (SP), CD9, CD10, CD13, CD29, CD31, CD34, CD44, CD117, CD133, FLT3, LNGFR, and FGFR3 (Caligaris-Cappio *et al*, 1985; Robinson *et al*, 1999; Erices *et al*, 2000; Singh *et al*, 2003; Kondo *et al*, 2004; Bobis *et al*, 2006; Jones *et al*, 2006; Small, 2008). We found that three rhabdomyosarcoma cell lines, KYM-1, RD, and A204, each included a small proportion of FGFR3-positive cells (1.6–2.6%) (Figure 1).

### The malignancy of KYM-1 and RD cells *in vivo* depends to a large extent on FGFR3-positive RICs

**Figure 2:**
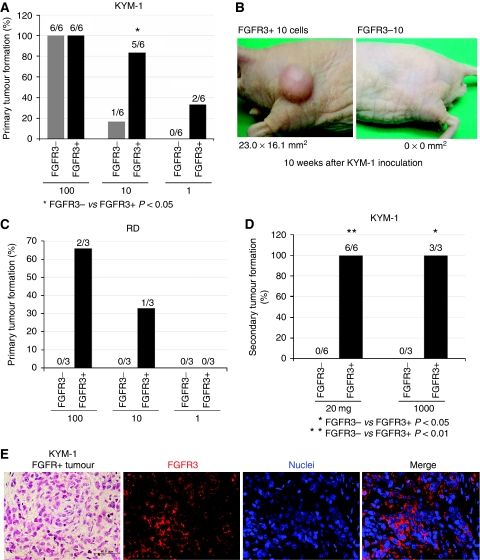
The malignancy of rhabdomyosarcoma cells *in vivo* depends to a large extent on the FGFR3-positive cells. FGFR3-dependent cell sorting was performed using immunomagnetic selection, with culture for one night to exclude dead cells by mechanical stress followed by inoculation of cells from either population interdermally into nude mice. (**A**) Primary tumour formation by KYM-1, FGFR3−, or FGFR3+ cells. After 8W, all mice inoculated with 100 KYM-1 cells had formed tumours. After 5W, in 5 out of 6 of 10 FGFR+ KYM-1 cells inoculated mice, there was tumour formation. In contrast, in 1 out of 6 of 10 FGFR− KYM-1 cells inoculated mice, there was tumour formation after 12W inoculation. Surprisingly, 2 out of 6 of only single FGFR3+ KYM-1 cell inoculated mice also exhibited tumour formation. In contrast, 0 out of 6 of single KYM-1 FGFR cell inoculated mice formed tumour after 12W inoculation. (**B**). Ten FGFR3-positive cells form tumour 8 weeks after inoculation. (**C**). Primary tumour formation by RD, FGFR3−, or FGFR3+ cells. After 6W, in 2 out of 3 of 100 FGFR+ RD cells inoculated mice, there was tumour formation. In contrast, in 0 out of 3 of 100 FGFR− RD cells inoculated mice, there was tumour formation after 12W inoculation. In addition, in 1out of 3 of 10 FGFR+ RD cells inoculated mice, there was tumour formation. In contrast, in 0 out of 3 of 10 FGFR− RD cells inoculated mice, there was tumour formation after 12W inoculation. (**D**) Secondary tumour formation by FGFR3− or FGFR3+ KYM-1 cells. We next performed serial transplantation. Small portions of formed tumour (20 mg) were excised and then inoculated into other nude mice. Six of six tumours formed by FGFR3+ KYM-1 cells inoculated into mice formed tumour. In contrast, none of six tumours formed by FGFR− KYM-1 cells inoculated into mice formed tumour after 12W inoculation. In addition, 3 out of 3 of 1000 cells prepared from FGFR3+ tumour inoculated mice formed tumour. In contrast, 0 out of 3 of 1000 cells prepared from FGFR− tumour inoculated mice formed tumour after 12W inoculation. (**E**) HE staining of tumour formed by FGFR3+ KYM-1cells. Immunohistochemical examination revealed that tumour formed by FGFR3+ KYM-1cells contains both FGFR3+ and FGFR3− cells (red: FGFR3, blue: Hoechst). ^*^ FGFR3− *vs* FGFR3+ *P*<0.05, ^*^^*^ FGFR3− *vs* FGFR3+ *P*<0.01.

To determine whether the subset defined by FGFR3 was enriched for RICs, we compared the abilities of FGFR3+ and FGFR3− rhabdomyosarcoma cells to initiate tumour formation *in vivo*. After 8W, all mice inoculated with 100 KYM-1 cells had formed tumours. After 5W, in 5 out of 6 of 10 FGFR+ KYM-1 cells inoculated mice, there was tumour formation. In contrast, in 1 out of 6 of 10 FGFR− KYM-1 cells inoculated mice, there was tumour formation. Surprisingly, 2 out of 6 of only single FGFR3+ KYM-1 cell inoculated mice also exhibited tumour formation. (Figure 2A). Next, we examined RD cells. After 6W, in 2 out of 3 of 100 FGFR+ RD cells inoculated mice, there was tumour formation. In contrast, in 0 out of 3 of 100 FGFR− RD cells inoculated mice, there was tumour formation after 12W inoculation. In addition, in 1 out of 3 of 10 FGFR+ RD cells inoculated mice, there was tumour formation. In contrast, in 0 out of 3 of 10 FGFR− RD cells inoculated mice, there was tumour formation (Figure 2C). We next performed serial transplantation. Small portions of formed tumour (20 mg) were excised and then inoculated into other nude mice. Six of six tumours formed by FGFR3+ cells inoculated into mice formed tumour. In contrast, none of six tumours were formed by FGFR− KYM-1 cells. In addition, 3 out of 3 of 1000 cells prepared from FGFR3+ tumour inoculated mice formed tumour (Figure 2D). In contrast, 0 out of 3 of 1000 cells prepared from FGFR− tumour formed tumour (Figure 2D). Immunohistochemical examination revealed that tumours formed by FGFR3+ KYM-1 cell contained both FGFR3+ cells and FGFR3− cells *in vivo* (Figure 2E).

### RICs express undifferentiated cell markers

**Figure 3:**
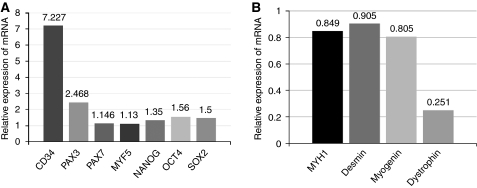
FGFR3-positive cells over-expressed undifferentiated cell genes. (**A**) As demonstrated by real-time PCR, FGFR3+ cells (RICs) over-express several undifferentiated cell marker genes compared with FGFR3− cells (**B**). Real-time PCR revealed that FGFR3+ cells (RICs) exhibited downregulation of several differentiated cell marker genes compared with FGFR3− cells. The comparative Ct (ΔΔCt) method was used to determine fold change in expression using *GAPDH* or *βII-microglobulin*. Each sample was run minimally at three concentrations in triplicate. The experiment was triplicate with similar results.

We next examined the expression of genes specific to skeletal muscle development or embryonic stem cells. RNA from FGFR3+ KYM-1 or FGFR3− KYM-1 cells was analysed by real-time PCR for *CD34*, *PAX3*, *PAX7*, *MYF5*, *NANOG*, *OCT4*, *SOX2*, *myosin heavy chain 1* (*MYH1*), *desmin*, *myogenin*, and *dystrophin*. Real-time PCR revealed that expression of *CD34* and *PAX3* in FGFR3+ cells was markedly increased by 7.23- and 2.47-fold, respectively. In addition, the expression of *PAX7*, *MYF5*, *NANOG*, *OCT3*, and *SOX2* was slightly increased by 1.15-, 1.13-, 1.35-, 1.56-, and 1.5-fold, respectively. On the other hand, the expression of the differentiated muscle markers *MYH1*, *desmin*, *myogenin*, and *dystrophin* was decreased to 0.85-, 0.91-, 0.81-, and 0.25-fold baseline levels, respectively (Figure 3).

### RICs can be maintained and enriched by bFGF

**Figure 4:**
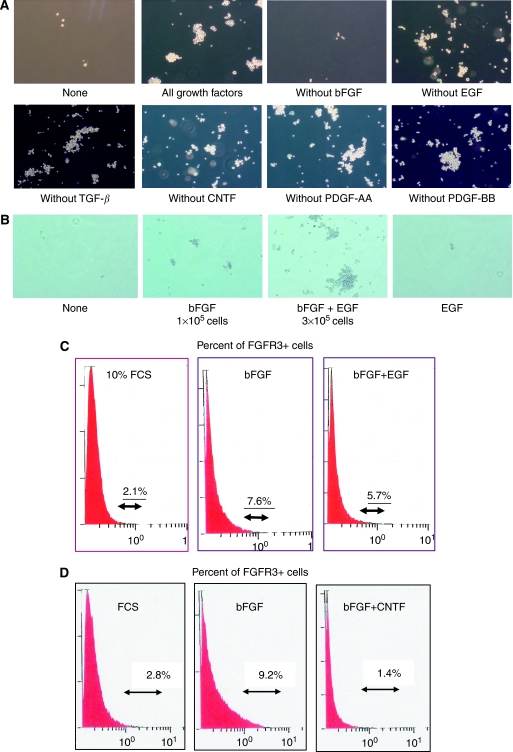
Increase in proportion of KYM-1 FGFR3-positive cells with bFGF. (**A**) KYM-1 cells were cultured in serum-free medium with or without growth factor cocktails. Cells were cultured for 3 weeks and then photographed. When cultured without bFGF, KYM-1 cells did not increase in proportion. (**B**) 1000 KYM-1 cells were cultured for 3 weeks in serum-free medium with bFGF, EGF, or both or neither and were then photographed. When cultured with bFGF, KYM-1 cells were maintained. Cell number was increased with bFGF plus EGF. (**C**) Proportion of FGFR3+ cells was analysed by flow cytometry. KYM-1 cells were cultured in FCS, bFGF, or bFGF plus EGF for 3 weeks. The proportion of FGFR3-positive RICs was increased by bFGF. When cultured with bFGF plus EGF, total number of cells was increased three-fold compared with bFGF alone. All experiments were repeated at least three times with similar results. (**D**) Proportion of FGFR3+ cells was analysed by flow cytometry. KYM-1 cells were cultured in FCS, bFGF, or bFGF plus CNTF for 3 weeks. When cultured with bFGF plus CNTF, the proportion of FGFR3+ cells was markedly decreased compared with bFGF alone or FCS. All experiments were repeated at least three times with similar results.

We then examined which factor(s) can maintain KYM-1 FGFR3+ cells in serum-free culture media. We tested bFGF, EGF, TGF-*β*1, CNTF, PDGF-AA, and PDGF-BB as candidates. These mitogens are important factors in maintaining many types of progenitor cells (Marmur *et al*, 1998; Kondo *et al*, 2004; Vallier and Pedersen, 2005). We first cultured unfractionated KYM-1 cells in serum-free culture medium alone or with a mixture of bFGF, EGF, TGF-*β*, CNTF, PDGF-AA, and PDGF-BB. KYM-1 cells could not grow without growth factors. On the other hand, this growth factor cocktail promoted KYM-1 cell growth. We next examined which mitogen is essential for KYM-1 cell growth, by withdrawing each mitogen individually. All culture conditions promoted KYM-1 growth but bFGF withdrawal (Figure 4A). These findings suggested that bFGF is essential for KYM-1 survival and proliferation. We cultured 1000 KYM-1 cells in each condition and counted 20 days after culture. Addition of EGF to serum-free culture medium with bFGF appreciably stimulated KYM-1 cell growth approximately three-fold, the same as neural progenitor cells (Figure 4B) (Kitchens *et al*, 1994). On the other hand, when cultured in serum-free culture medium with EGF alone, KYM-1 cells could not survive. We then stained cells with anti-FGFR3 antibody and analysed them by flow cytometry. When cultured in serum-free medium with bFGF, FGFR3+ cells were maintained, and their proportion increased to 7.6–9.2%. In addition, when cultured in both bFGF and EGF, FGFR3+ cells increased to 4.2–6.0 % and total cell number increased three-fold (Figure 4C). We next examined which factor prevents expansion of FGFR3+ cells. When KYM-1 cells were cultured with bFGF plus CNTF, CNTF reduced the proportion of FGFR3+ cells by approximately 15% (Figure 4D), although CNTF did not affect the total number of KYM-1 cells. These findings suggest that RICs can be maintained and increased in bFGF alone and that a combination of bFGF and EGF can increase cell numbers. On the other hand, CNTF decreased the proportion of RICs.

### FGFR3 was upregulated in rhabdomyosarcomapatient biopsy specimens

**Figure 5:**
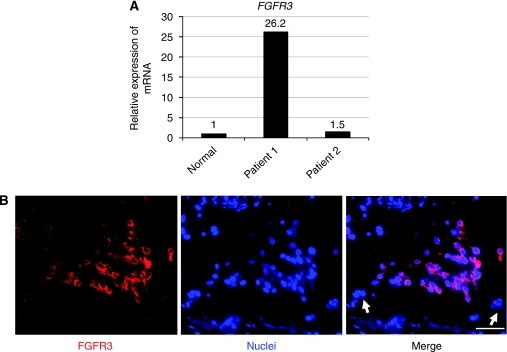
FGFR3 is over-expressed in rhabdomyosarcoma patient specimens. (**A**) We obtained two biopsy specimens of human rhabdomyosarcoma from primary lesions. Pathological examination revealed that patient 1 had embryonal rhabdomyosarcoma and patient 2 had alveolar rhabdomyosarcoma. Real-time PCR revealed that the amount of FGFR mRNA in the embryonal rhabdomyosarcoma biopsy sample was more than that in the normal skeletal muscle or alveolar rhabdomyosarcoma sample. (**B**) Immunohistochemistry revealed that FGFR3 was expressed in a portion of rhabdomyosarcoma patient 1 biopsy specimens. Arrows indicate FGFR3-negative cells. Immunohistochemical examination showed that 11.2±2.8% cells were positive for FGFR3.

We next examined the expression of FGFR3 in patient biopsy specimens. Real-time PCR revealed that FGFR3 was upregulated in embyonal rhabdomyosarcoma patient biopsy specimens (Figure 5A). Immunohistochemical examination revealed that a portion of rhabdomyosarcoma cells expressed FGFR3. The intensity of FGFR3 expression differed among rhabdomyosarcoma cells (Figure 5B).

## Discussion

Although there is an expanding literature supporting the existence of cancer stem cells, important caveats of these studies continue to provoke debate. The current definitive test for a cancer stem cell is the capacity to propagate tumours as xenografts in immunocompromised mice (Clarke *et al*, 2006). We have described here the isolation of a highly tumourigenic subpopulation of cells from human rhabdomyosarcoma cell lines in accord with terminology. To our knowledge, this is the first isolation of malignant progenitors from human rhabdomyosarcoma to be described.

Initially, to identify candidate RICs, we used a side population method as reported earlier (Kondo *et al*, 2004; Setoguchi *et al*, 2004). We detected approximately 1–3% SP cells among KYM-1 cells. We sorted SP and non-SP cells and then inoculated them into nude mice subcutaneously, but could not detect differences between them in tumourigenisity (data not shown). We next examined CD133, which has been reported to be a cancer stem cell marker (Singh *et al*, 2003; Hermann *et al*, 2007; Ricci-Vitiani *et al*, 2007; Chearwae and Bright, 2008; Mizrak *et al*, 2008). KYM-1 cells also included a small proportion of CD133-positive cells. We sorted CD133+ and CD133− cells and then inoculated them into nude mice subcutaneously, but found no differences between them in tumourigenisity (data not shown). In addition, we were unable to identify subpopulations by other undifferentiated cell markers. In our study, RICs were enriched in rhabdomyosarcoma subpopulations defined by FGFR3 alone. These findings suggest that sarcomas may differ from other epithelial malignancies, including cancers of the breast, head and neck, lung, pancreas, colon, and prostate (Al-Hajj *et al*, 2003; Collins *et al*, 2005; Fang *et al*, 2005; Kim *et al*, 2005; Dalerba *et al*, 2007; Li *et al*, 2007; Prince *et al*, 2007). It has been reported that FGFR3 is expressed in human muscle from 11 weeks of gestation and is decreased in adult muscle (Sogos *et al*, 1998). Muscle stem cells (muscle satellite cells) express FGFR3 whereas muscle-derived fibroblasts do not (Sheehan and Allen, 1999). These findings suggest that FGFR3 is expressed not only in RICs but also in muscle stem cells. In addition to exhibiting aggressive tumourigenisity, RICs expressed stem cell markers intensely with fewer markers of differentiation. These findings suggest that RICs have the characteristics of undifferentiated cells. In particular, RICs upregulated *CD34* and that downregulated *dystrophin*. These genes are muscle cell linage specific. These findings suggest that RICs are already to some extent committed to the muscle cell linage from more undifferentiated stages such as mesenchymal stem cells.

Rhabdomyosarcoma is composed of embryonal and alveolar subtypes. KYM-1 and RD is established from embryonal rhabdomyosarcoma (McAllister *et al*, 1969; Sekiguchi *et al*, 1985). The subtype of A204 was not described in article of cell line establishment (Giard *et al*, 1973). Embryonal rhabdomyosarcoma contain primitive undifferentiated round cells (Gallego Melcon and Sanchez de Toledo Codina, 2007). Consistent with these findings, we showed that embryonal rhabdomyosarcoma cell lines contain undifferentiated RICs. In addition, real-time PCR revealed that the amount of FGFR mRNA in the embryonal rhabdomyosarcoma biopsy sample was more than that in the normal skeletal muscle or alveolar rhabdomyosarcoma sample.

Mammals have four FGFR tyrosine kinase genes (FGFR1–4) (Eswarakumar *et al*, 2005). FGFRs are composed of an extracellular ligand-binding domain, a transmembrane domain, and a split cytoplasmic tyrosine kinase domain. In this study, we examined only FGFR3. Whether related members of the FGFR family are markers of RICs requires further study. We found that bFGF could maintain and expand RICs. It has been reported that bFGF promotes proliferation and inhibits differentiation of muscle satellite cells (Guthridge *et al*, 1992; Lefaucheur and Sebille, 1995). The bFGF binds to FGFR1, FGFR2, FGFR3, and FGFR4. The binding of bFGF to FGFR3 activate FGF signalling pathway (Ornitz and Leder, 1992; Maric *et al*, 2007). These data suggest that FGFR3 is not only a cell surface marker for RICs but also mediates signals important for RICs maintenance and proliferation. In addition, we found that CNTF reduced the proportion of RICs. There is increasing evidence that chemotherapy and radiation can each efficiently eradicate the majority of malignant cells within neoplastic lesions. However, these regimens frequently fail to eliminate a minor subpopulation of resistant cancer stem cells (Trumpp and Wiestler, 2008). Inhibition of FGFR3 signalling or activation of CNTF signalling might thus be a good candidate for anti-cancer stem cell therapy for rhabdomyosarcoma.

Recent studies have suggested that FGFR3 has a significant function in the pathogenesis and progression of some malignancies including thyroid carcinoma, bladder carcinoma, multiple myeloma, and peripheral T-cell lymphoma (Cappellen *et al*, 1999; Onose *et al*, 1999; Kastrinakis *et al*, 2000; Yagasaki *et al*, 2001; Wolff *et al*, 2005). Whether FGFR3 is a marker of TICs in these malignancies requires further study.

In our study, 33% of single KYM-1 RICs formed tumours. This TIC frequency is somewhat higher than previously reported for other TICs (Singh *et al*, 2003; Hermann *et al*, 2007; Ricci-Vitiani *et al*, 2007; Mizrak *et al*, 2008). These more strong tumourigenic RICs may be more useful than other TICs for examining the molecular mechanisms of tumour initiation, proliferation, anti-apoptotic capacity, and metastasis. Although RICs are enriched in the rhabdomyosarcoma subpopulations defined by FGFR3, not every FGFR3+ cell is an RIC, as 67% of purified single RICs did not form tumours. Quintana *et al* reported that frequency of tumourigenisity in mice depends to a large extent on the status of immunodeficiency (Quintana *et al*, 2008). When melanoma cells were transplanted into NOD/SCID mice, 1 in 111 000 cells formed tumour. When transplanted into highly immunocompromised NOD/SCID interleukin-2 receptor *γ* chain null mice, 27% of single cells formed tumours. The tumourigenisity of RICs might vary depending on the experimental conditions used, such as the tissue site of xenotransplantation, or differences among recipient immunodeficient mice.

In summary, we identified FGFR3-positive RICs in human rhabdomyosarcoma cell lines. RICs were more strongly tumourigenic than other previously reported TICs. Our easy method of preparing RICs may prove useful for further exploration of pathogenesis of rhabdomyosarcoma and molecular characterisation of cancer stem cells.
